# Robotic infraclavicular approach for minimally invasive neck dissection: an evaluation of safety, oncologic efficacy, and learning curve dynamics in head and neck cancer surgery

**DOI:** 10.3389/fonc.2026.1806241

**Published:** 2026-08-03

**Authors:** Athira Ramakrishnan, Sandeep P. Nayak, Ameenuddin Khan, Abhilasha Sadhoo, Bharath Gangadhara, Sreekanth Reddy, Devaprasad Munisiddaiah, Suraga Belakawadi, P. Vinodhini, Vidyashri Biral, Keerthi Ranganathan

**Affiliations:** 1 Department of Surgical Oncology, MACS-Renova Oncology Institute, KIMS Hospitals (Krishna Institute of Medical Sciences), Bengaluru, Karnataka, India; 2 Department of Surgical Oncology, Adyar Cancer Institute, Chennai, Tamil Nadu, India

**Keywords:** CUSUM algorithm, head and neck (H&N) cancer, infraclavicular approach, minimally invasive techniques, robotic neck dissection (ND)

## Abstract

**Introduction:**

Conventional open lateral neck dissection in head and neck cancer is associated with visible cervical scarring and procedure-related morbidity. Although robotic remote-access approaches improve cosmesis, many require larger incisions or provide limited access to certain cervical nodal levels. The Robotic Infraclavicular Approach for Minimally Invasive Neck Dissection (RIA-MIND) was developed to enable comprehensive cervical lymphadenectomy through smaller, cosmetically concealed infraclavicular incisions.

**Methods:**

We performed a retrospective cohort study of 62 patients who underwent robotic neck dissection using the RIA-MIND technique between July 2018 and May 2025 at a tertiary oncology center. Clinicopathological, perioperative, and oncological data were analyzed, including nodal yield, preservation of key cervical structures, postoperative complications, adjuvant treatment, and recurrence. Learning curve assessment was performed using cumulative sum (CUSUM) analysis of operative (console) time, ipsilateral nodal yield, and perioperative complications.

**Results:**

The tongue was the most common primary tumor site (67.7%), and squamous cell carcinoma constituted 98.4% of cases. Median ipsilateral and contralateral nodal yields were 25 (IQR 20–35) and 17 (IQR 12.25–21.25), respectively. The spinal accessory nerve and internal jugular vein were preserved in 98.4% of patients, while the sternocleidomastoid muscle was preserved in 53.2%. Postoperative complications were infrequent, with 87.1% of patients experiencing no documented complications. Recurrence was observed in 5 patients (8.1%), all of which were local recurrences; no regional neck recurrence was observed. CUSUM analysis demonstrated a structured learning curve with progressive improvement in console efficiency, stabilization of ipsilateral nodal yield, and maintenance of perioperative safety over time.

**Conclusion:**

RIA-MIND is a feasible and safe remote-access robotic technique for unilateral and bilateral neck dissection in selected head and neck cancers, providing satisfactory nodal yield with low perioperative morbidity and favorable early oncologic outcomes. The structured learning curve supports its technical reproducibility; however, further prospective comparative studies with longer follow-up are required to establish long-term oncologic, functional, and cosmetic outcomes.

## 1 Introduction

Asia accounts for approximately 65.8% of the global burden of head and neck cancers and nearly 72% of associated deaths (1). In this context, and amid the high incidence of head and neck cancers in India, management of the cervical lymph nodes (LN) remains a critical component of treatment (2). Neck metastasis is one of the strongest predictors of survival, and 20–30% of clinically N0 patients may harbor occult nodal disease (3). Conventional open lateral neck dissection (OLND) provides excellent exposure for clearance of nodal levels I–V; however, it requires a cervical incision that results in a conspicuous scar and may cause functional morbidity, particularly shoulder dysfunction related to spinal accessory nerve (SAN) traction. These cosmetic and functional drawbacks negatively affect quality of life, even when oncologic outcomes are satisfactory (4).

To avoid visible neck scarring, several minimally invasive “scarless-at-the-neck” approaches have been developed, including transaxillary, retroauricular, bilateral axillary, and transoral techniques. While these endoscopic and robotic methods improve cosmesis and visualization, each has inherent limitations. Transaxillary and chest-based approaches are restricted by the clavicle, limiting access to levels IV and V, whereas transoral routes inadequately expose levels Ia and II. Moreover, many robotic approaches require long incisions (7–12 cm), specialized retractors, or are unsuitable for bilateral neck dissection (4).

Our group has played a pivotal role in the development of minimally invasive neck dissection (MIND). In 2015, we first demonstrated the feasibility of gas-insufflated endoscopic MIND via a subclavian approach using standard laparoscopic equipment, achieving excellent cosmetic outcomes without compromising oncologic safety (5). This was followed by a 3-year retrospective series of 45 patients that established the reproducibility, safety, and efficacy of the technique (6). Subsequently, a systematic review and meta-analysis further validated MIND as a safe alternative to conventional OLND, with advantages in intraoperative blood loss, incision length, drainage parameters, and hospital stay (7). More recently, we reported the first description of the robotic infraclavicular approach for minimally invasive neck dissection (RIA-MIND), demonstrating its technical feasibility and early safety profile in a pilot case report (8).

Notably, despite these advances, no prior studies have systematically evaluated the learning curve for minimally invasive or robotic infraclavicular neck dissection techniques. Building on this background, the present study was designed to assess the feasibility, safety, nodal yield, functional outcomes, and learning curve of RIA-MIND in a larger cohort of patients with head and neck cancers.

## 2 Patients and methods

### 2.1 Study design and patient selection

#### 2.1.1 Study design

**Figure 1:**
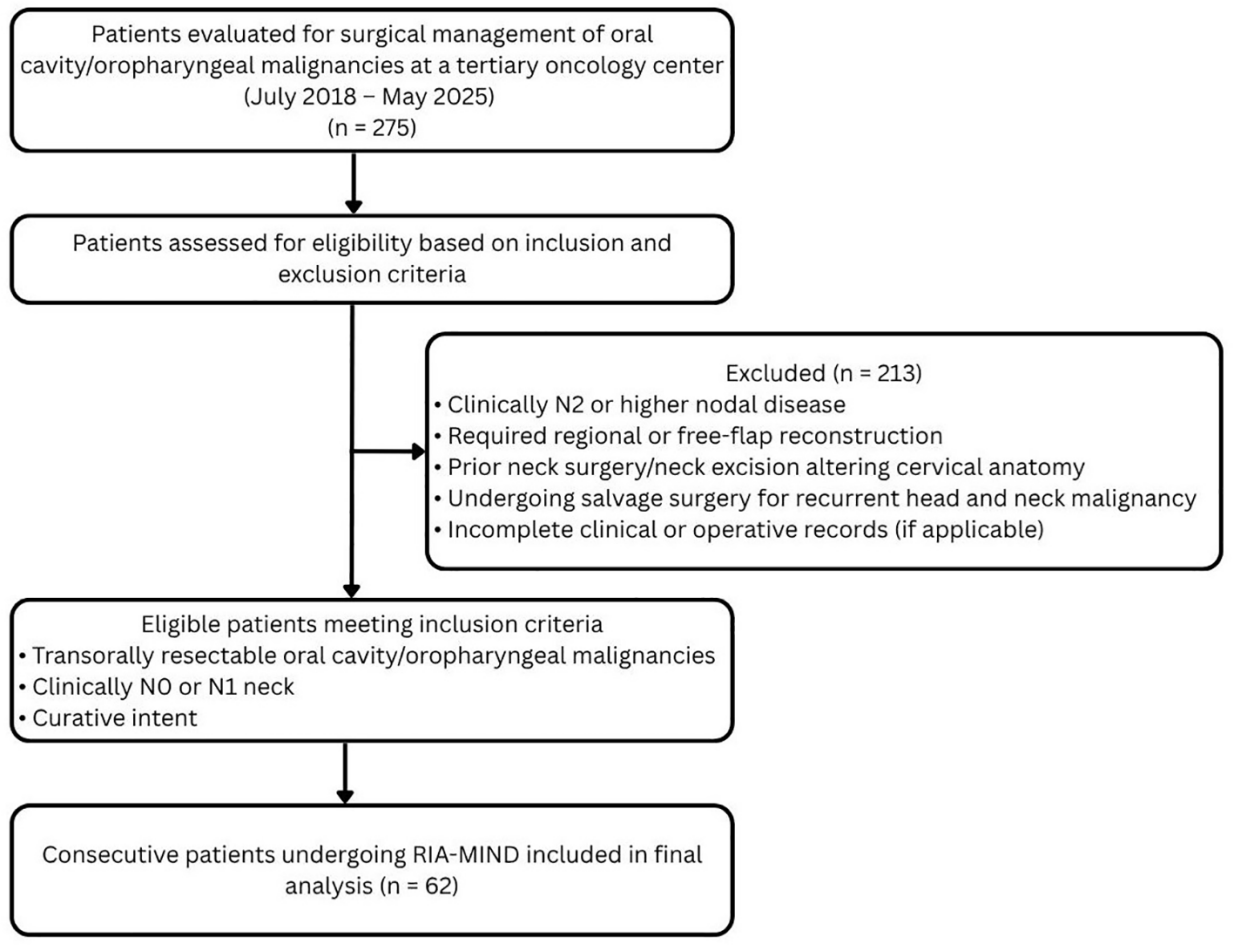
STROBE flow diagram of patient selection.

This retrospective cohort study was conducted at a tertiary oncology centre and included consecutive patients who underwent robotic neck dissection using the RIA-MIND technique for histologically confirmed head and neck malignancies between July 2018 and May 2025. The primary objective of the study was to assess the feasibility, peri-operative safety, and immediate postoperative outcomes of the RIA-MIND approach in a consecutive institutional series; it was not designed as a survival analysis study. Patient selection is summarized in the STROBE flow diagram (**Figure 1**). Ethical clearance was obtained from the Institutional Ethics Committee at Fortis Hospital. The study adhered to the STROBE guidelines and the Declaration of Helsinki (2013). All data were anonymised; no additional interventions were performed for research purposes; and written informed consent was obtained from all patients as part of routine clinical care.

#### 2.1.2 Inclusion criteria

Patients with histologically confirmed oral cavity or oropharyngeal malignancies who underwent robotic neck dissection using the RIA-MIND approach with curative intent were included. Eligible patients had transorally resectable primary tumors with clinically N0 or N1 nodal disease and did not require regional or free-flap reconstruction.

#### 2.1.3 Exclusion criteria

Patients with clinically N2 or higher nodal disease, those requiring regional or free-flap reconstruction, patients undergoing salvage surgery for recurrent head and neck malignancy, those with prior neck surgery altering cervical anatomy, and patients with incomplete clinical or operative data were excluded.

### 2.2 Surgical approach

The surgical technique used in this study is based on our previously published description of RIA-MIND, which demonstrated the feasibility and safety of this remote-access minimally invasive neck dissection method (8). All procedures were performed by a single surgeon using the RIA-MIND technique. Patients were positioned supine with a shoulder roll to achieve neck extension and rotated to the contralateral side of the dissection. Three access ports were placed below the clavicle: the medial port 1.5 cm inferior to the clavicle, a second port below the midpoint of the clavicle, and a lateral port positioned medial to the acromion, also 1.5 cm below the clavicle. The central port incision was created first, and a working space was developed above the pectoralis muscle fascia and across the clavicle, extending approximately one centimetre beyond it. Similar dissection was carried laterally to create space for the remaining ports, and an inferolateral soft-tissue pocket was developed for the assistant port. All ports were secured, and the da Vinci robotic system was docked with the camera in the central port, monopolar scissors in the right arm, and bipolar forceps in the left arm.

A subplatysmal flap was elevated cranially after identification of the platysma, extending superiorly to the mandible, medially to the midline, and posteriorly to the anterior border of the trapezius, replicating the exposure of a conventional OLND.

### 2.3 Data collection

Clinical, operative, and pathological data were extracted from institutional electronic medical records and surgical databases. Variables collected included demographic characteristics; tumour site and histopathology; neoadjuvant treatment; details of the robotic surgical technique; intraoperative findings; postoperative recovery parameters; all documented postoperative complications identified through review of operative notes, inpatient records, and follow-up documentation; time to stoma closure, where applicable; and long-term oncological outcomes.

## 3 Statistical analysis

Basic descriptive statistics were performed using Epi Info™ version 7 and Google Colab. Continuous variables were summarised using means, standard deviations, medians, and percentiles, while categorical variables were reported as frequencies and percentages.

Learning curve analysis was performed using the cumulative sum (CUSUM) methodology to assess operative performance and safety across sequential RIA-MIND procedures. Chronologically ordered cases were analyzed.

A mean-referenced (classical) CUSUM approach was applied to continuous performance variables, including operative (console) time and ipsilateral nodal yield, to evaluate trends in technical efficiency and oncological adequacy. For these parameters, the cumulative deviation of each case from the overall cohort mean was plotted sequentially. Learning phases were initially identified by visual inspection of the CUSUM trajectories. They were subsequently validated using segmented (piecewise) linear regression, enabling objective identification of inflection points corresponding to learning, transition, and proficiency phases.

To assess procedural safety, a binary (Bernoulli) CUSUM analysis was performed for perioperative complications, with each case classified as having or not having a complication. This approach was used to monitor deviations in the incidence of complications over time relative to the expected event rate.

## 4 Results

### 4.1 Demographic and clinical profile

A total of 62 patients underwent surgery using the RIA-MIND approach. The mean age of the cohort was 57.03 ± 13.06 years, with 36 males (58.06%) and 26 females (41.94%). The mean body mass index (BMI) was 26.02 ± 3.74 kg/m². Most patients were overweight (43.54%) or had a healthy BMI (35.48%). Comorbidities were common, with hypertension being the most frequently observed condition (40.32%), followed by diabetes mellitus (20.97%) and hypothyroidism (8.06%) (**Table 1**).

**Table 1:** Demographic and pre-operative profile.

Parameter	Category/statistic	RIA-MIND (N = 62)
Age (years)	Mean ± SD	57.03 ± 13.06
Gender (n, %)	Male	36 (58.06%)
	Female	26 (41.94%)
BMI (kg/m²)	Mean ± SD	26.02 ± 3.74
	Underweight (n, %)	2 (3.23%)
	Healthy (n, %)	22 (35.48%)
	Overweight (n, %)	27 (43.54%)
	Obese (n, %)	9 (14.52%)
	Not captured in the study database (n, %)	2 (3.23%)
Comorbidities (n, %)	Bronchial Asthma	3 (4.84%)
	Diabetes Mellitus	13 (20.97%)
	Hepatitis B Surface Antigen	1 (1.61%)
	Hepatitis C Virus	2 (3.23%)
	Hypertension	25 (40.32%)
	Hypothyroidism	5 (8.06%)
	Schizophrenia	1 (1.61%)
	Seizure Disorder	1 (1.61%)
	Vitiligo	1 (1.61%)

RIA-MIND, Robotic Infraclavicular Approach for Minimally Invasive Neck Dissection; SD, Standard Deviation; BMI, Body mass index; IQR, Interquartile Range.

### 4.2 Tumor characteristics

The tongue was the predominant site of tumor, accounting for 67.75% of cases, followed by the buccal mucosa in 17.75% of patients. Histopathologically, squamous cell carcinoma constituted the majority of tumors (98.39%), with only one case of mucoepidermoid carcinoma. Most patients presented with localized disease (62.90%), while 37.10% had regional involvement.

Unilateral RIA-MIND was performed in 77.42% of patients, whereas bilateral RIA-MIND was performed in 22.58%. Bilateral neck dissection was performed for tongue cancers with a depth of invasion greater than 1 cm and for tumors located at or crossing the midline (**Table 2**).

**Table 2:** Tumor characteristics and peri-operative details.

Parameter	Category/statistic	RIA-MIND (N = 62)
Tumor Site (n, %)	Alveolus	1 (1.61%)
	Buccal mucosa	11 (17.75%)
	Floor of mouth	2 (3.23%)
	Hard palate	1 (1.61%)
	Lip	1 (1.61%)
	Maxilla	1 (1.61%)
	Retromolar trigone	1 (1.61%)
	Scalp	1 (1.61%)
	Tongue	42 (67.75%)
	Tonsil	1 (1.61%)
Histopathology (n, %)	Mucoepidermoid carcinoma	1 (1.61%)
	Squamous cell carcinoma	61 (98.39%)
Extent of Disease (n, %)	Localized	39 (62.90%)
	Regional	23 (37.10%)
Laterality (n, %)	Unilateral	48 (77.42%)
	Bilateral	14 (22.58%)
Operative Time (Median, IQR)	Surgical Time	360.00 (300.00 - 428.75)
	Console Time	177.00 (150.75 - 220.00)
	Unilateral Procedures	152.50 (104.00–212.50)
	Bilateral Procedure	220.00 (173.50–248.25)
Lymph Nodes (Median, IQR)	Total Ipsilateral Nodes	25.00 (20.00 - 35.00)
	Positive Ipsilateral Nodes	2.00 (1.00 - 2.00)
	Total Contralateral Nodes	17.00 (12.25 - 21.25)
	Positive Contralateral Nodes	1.00 (1.00 - 1.00)
Neck Structure Preservation (n, %)	SCM	33 (53.23%)
	SAN	61 (98.39%)
	IJV	61 (98.39%)

RIA-MIND, Robotic Infraclavicular Approach for Minimally Invasive Neck Dissection; SD, Standard Deviation; IQR, Interquartile Range; SCM, Sternocleidomastoid Muscle; SAN, Spinal Accessory Nerve; IJV, Internal Jugular Vein.

### 4.3 Peri-operative details

The median operative time for the RIA-MIND procedure was 360.00 minutes (IQR 300.00–428.75), with a median console time of 177.00 minutes (IQR 150.75–220.00). The median operative time was 152.50 minutes (IQR 104.00–212.50) for unilateral procedures and 220.00 minutes (IQR 173.50–248.25) for bilateral procedures. LN dissection yielded a median of 25 ipsilateral nodes (IQR 20–35) and 17 contralateral nodes (IQR 12.25–21.25). The SAN and internal jugular vein (IJV) were preserved in 98.39% of patients, and the sternocleidomastoid muscle (SCM) in 53.23% (**Table 2**).

### 4.4 Post-operative outcomes

In terms of pathological staging, T2 tumors were the most common (41.94%), followed by T1 tumors (38.71%). Nodal staging showed that 62.90% of patients were node-negative (N0). Negative (R0) resection margins were achieved in all patients. Postoperative complications were uncommon; 87.10% of patients experienced none. The most frequent complications included chyle leak and oral bleeding (each 3.23%), which were managed postoperatively. Lymphovascular invasion (LVI) was present in 8.06% of patients, perineural invasion (PNI) in 11.29%, and extranodal extension (ENE) in 3.23% of patients (**Table 3**).

**Table 3:** Post-operative findings and outcomes.

Parameter	Category/statistic	RIA-MIND (N = 62)
TNM staging		
T Stage	Tx	3 (4.84%)
	T1	24 (38.71%)
	T2	26 (41.94%)
	T3	5 (8.06%)
	T4a	3 (4.84%)
	Lost to Follow-up (n, %)	1 (1.61%)
N Stage	N0	39 (62.90%)
	N1	5 (8.06%)
	N1a	1 (1.61%)
	N1b	1 (1.61%)
	N2	3 (4.84%)
	N2a	1 (1.61%)
	N2b	6 (9.68%)
	N2c	4 (6.45%)
	N3	1 (1.61%)
	Lost to Follow-up (n, %)	1 (1.61%)
Complications (n, %)	Chyle Leak	2 (3.23%)
	Hypoglossal Nerve Palsy	1 (1.61%)
	IJV Injury	1 (1.61%)
	Oral Bleeding	2 (3.23%)
	Post-operative Infection	1 (1.61%)
	Vocal Cord Paresis	1 (1.61%)
	Nil	54 (87.10%)
Lymphovascular invasion	Present	5 (8.06%)
	Absent	57 (91.94%)
Perineural invasion	Present	7 (11.29%)
	Absent	55 (88.71%)
Extranodal Extension (n, %)	Present	2 (3.23%)
	Absent	60 (96.77%)
Recurrence pattern	Local (n, %)	5 (8.06%)
Radiotherapy	Received	25 (40.32%)
	Didn’t receive	37 (59.68%)
Chemoradiotherapy	Received	7 (11.29%)
	Didn’t receive	55 (88.71%)
Outcome Status	Alive	47 (75.81%)
	Dead	9 (14.52%)
	Lost to follow-up	6 (9.67%)

RIA-MIND, Robotic Infraclavicular Approach for Minimally Invasive Neck Dissection; IJV, Internal Jugular Vein; IQR, Interquartile Range.

### 4.5 Adjuvant therapy and recurrence

Adjuvant radiotherapy was administered to 40.32% of patients, while 11.29% underwent concurrent chemoradiotherapy. Recurrence was observed in 5 patients (8.06%), all of whom had local recurrences. No regional (neck) recurrences were identified during the follow-up period (**Table 3**).

### 4.6 CUSUM learning curve

#### 4.6.1 Mean-referenced CUSUM analysis of console time

**Figure 2:**
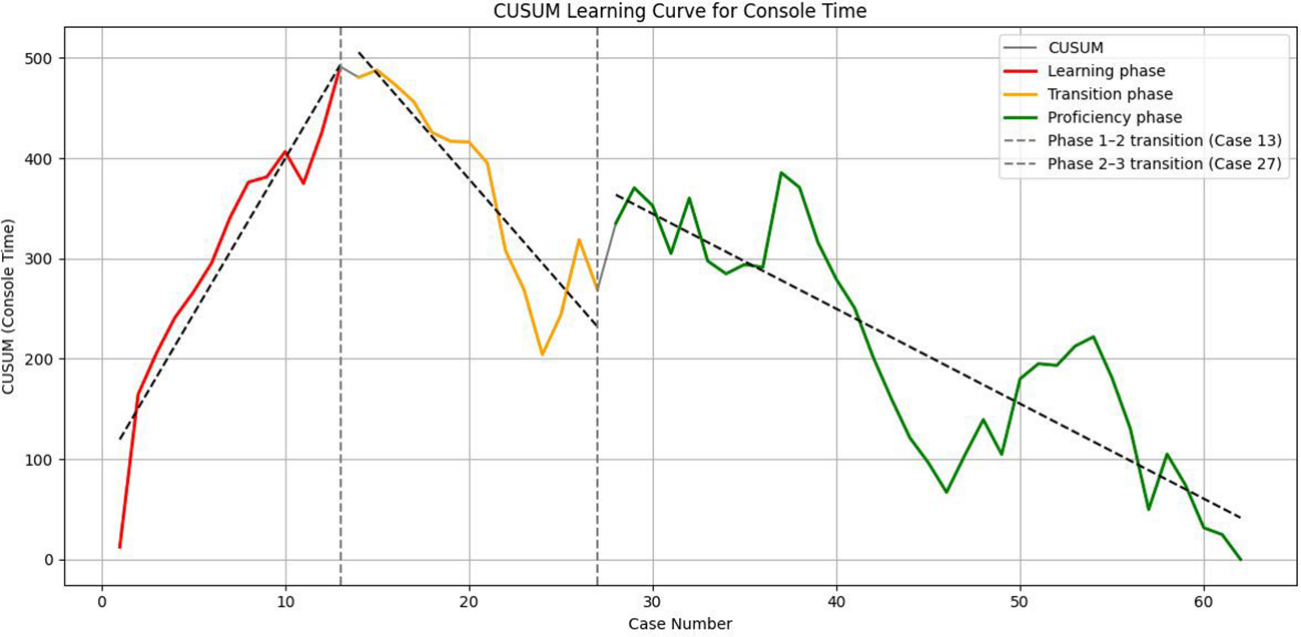
Mean-referenced CUSUM learning curve for operative (console) time in RIA-MIND. CUSUM plot demonstrating the sequential deviation of console time from the overall cohort mean across 62 consecutive RIA-MIND procedures. Segmented regression was used to demarcate learning, transition, and proficiency phases, providing an objective representation of performance progression over time.

Mean-referenced CUSUM analysis of console time demonstrated a clear learning curve across sequential RIA-MIND procedures (**Figure 2**). The CUSUM curve showed a progressive upward trend during the initial cases, indicating console times above the cohort mean during the learning phase. This was followed by a transitional phase characterized by a gradual downward trajectory, reflecting improving console efficiency. In the latter cases, the curve showed a sustained decline toward baseline, consistent with further reduction and stabilization of console time as surgical experience increased. Segmented regression identified two inflection points at cases 13 and 27, corresponding to distinct learning (cases 1–13), transition (cases 14–27), and proficiency (cases ≥28) phases. These findings suggest progressive acquisition of robotic console proficiency, with marked improvement in efficiency after the initial learning period.

#### 4.6.2 Mean-referenced CUSUM analysis of ipsilateral nodal yield

**Figure 3:**
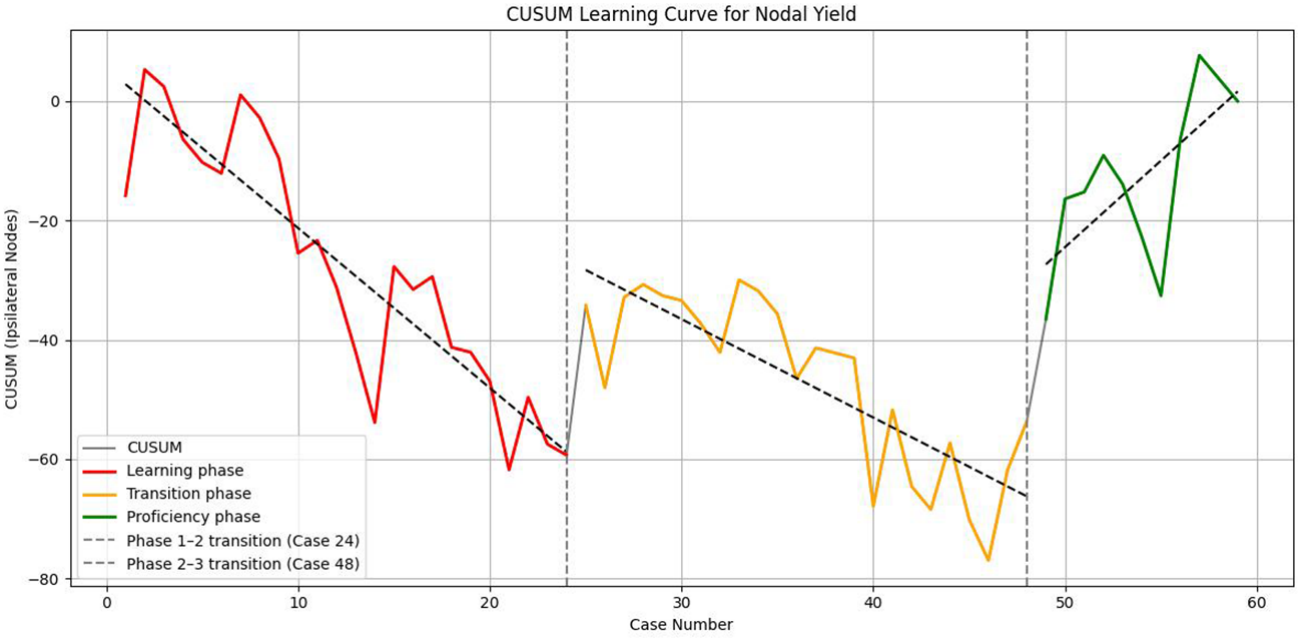
Mean-referenced CUSUM learning curve for ipsilateral nodal yield in RIA-MIND. CUSUM plot demonstrating the sequential deviation of ipsilateral nodal yield from the overall cohort mean across 62 consecutive RIA-MIND procedures. Segmented regression was used to demarcate learning, transition, and proficiency phases, providing an objective representation of performance progression over time.

Mean-referenced CUSUM analysis of ipsilateral nodal yield demonstrated a distinct learning pattern over the operative sequence (**Figure 3**). Segmented regression identified three distinct phases comprising a learning phase (cases 1–24), a transition phase (cases 25–48), and a proficiency phase (cases ≥49). While early cases demonstrated cumulative negative deviation from the cohort mean, the proficiency phase showed a sustained upward CUSUM trend, indicating consistent retrieval of nodal counts exceeding the cohort mean and supporting progressive stabilization of oncologic adequacy with increasing surgical experience.

#### 4.6.3 Binary (Bernoulli) CUSUM analysis for peri-operative complications

**Figure 4:**
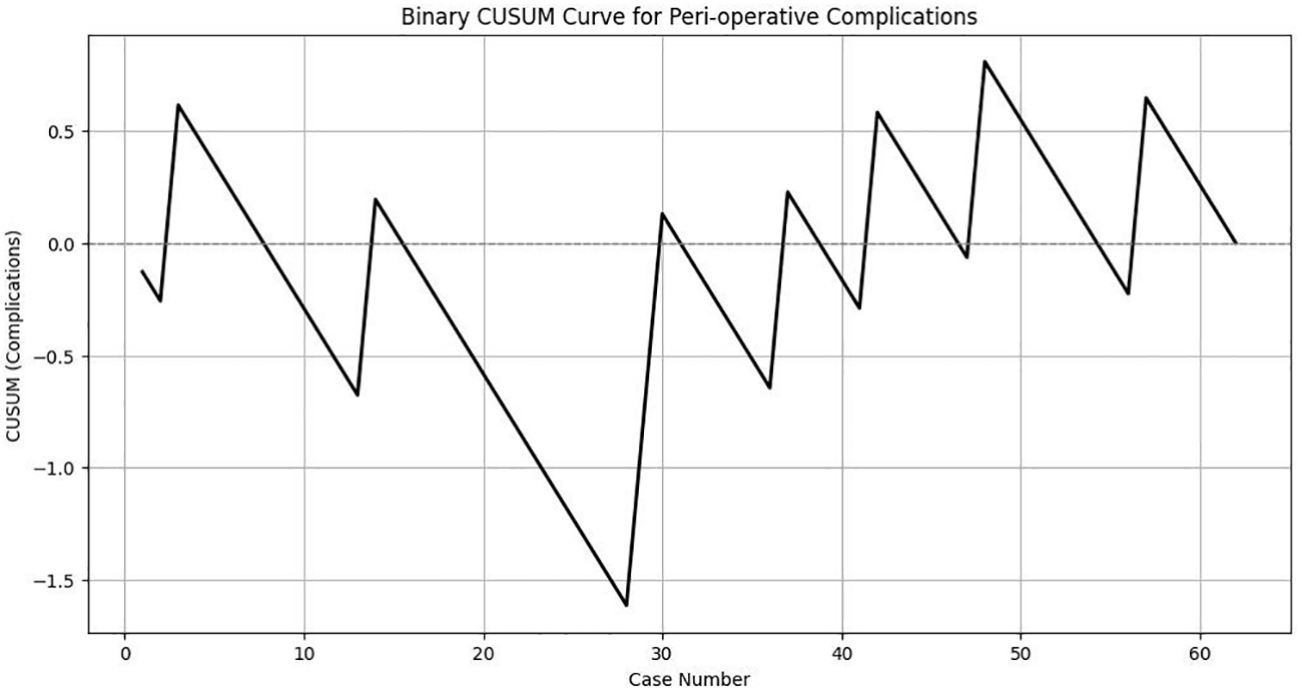
Binary (Bernoulli) CUSUM learning curve for peri-operative complications in RIA-MIND. CUSUM plot demonstrating the sequential deviation of peri-operative complication rate from the overall cohort mean across 62 consecutive RIA-MIND procedures.

Binary (Bernoulli) CUSUM analysis for peri-operative complications demonstrated early fluctuations around the expected complication rate, followed by stabilization without a sustained upward trend across subsequent cases (**Figure 4**).

## 5 Discussion

### 5.1 Innovation and technical rationale of RIA-MIND

The present study demonstrates that RIA-MIND is a feasible and safe alternative to conventional OLND and previously described minimally invasive techniques. Building on our earlier experience with gas-insufflated endoscopic MIND (5–8), this study represents the first expanded clinical series evaluating RIA-MIND as a comprehensive approach capable of clearing nodal levels I–V with minimal morbidity.

A key innovation of RIA-MIND lies in its ability to overcome the anatomic limitations inherent in other remote-access techniques. While transaxillary, retroauricular, transoral, and hybrid approaches offer cosmetic benefits, they are often restricted in accessing nodal levels II, IV, and V, or require long incisions and specialized retractors (4). In contrast, RIA-MIND provides a stable inferior working corridor through small infraclavicular incisions, allowing consistent exposure of all cervical nodal levels. This configuration improves visualization and instrument maneuverability while preserving a “scarless-at-the-neck” cosmetic outcome.

In the context of other minimally invasive approaches, these findings are particularly noteworthy. Transaxillary, retroauricular, and hybrid techniques typically require larger incisions (7–12 cm) and often face difficulty in accessing level V or the lower neck due to clavicular obstruction (9–11). Endoscopic chest approaches have difficulties in reaching levels IV–V, whereas transoral approaches may provide inadequate exposure of levels I and II due to the acute operative angle (12). In contrast, RIA-MIND offers a direct inferior trajectory that overcomes these anatomical constraints and enables comprehensive unilateral or bilateral neck dissection, an advantage not consistently achievable with most alternative minimally invasive techniques.

The cosmetic and functional advantages of RIA-MIND are especially relevant in a population where visible neck scarring and postoperative dysfunction can significantly affect quality of life. By placing incisions infraclavicularly, the technique avoids the social and psychological burden associated with prominent cervical scars while allowing early recovery and timely initiation of adjuvant therapy (13).

### 5.2 Safety and functional preservation

Perioperative morbidity was low, with 87.10% of patients experiencing no complications. Chyle leak occurred in 3.23%, within reported ranges for OLND (2–8%) (14, 15). Nerve-related complications were uncommon, with hypoglossal nerve palsy and vocal cord paresis each occurring in 1.61%, lower than rates reported in conventional series (≈3–4%) (16). Vascular injury was rare, with a single IJV injury (1.61%), contrasting with OLND series reporting multiple vascular, lymphatic, and nerve injuries across intraoperative and delayed periods (17). Other complications, including oral bleeding (3.23%) and postoperative infection (1.61%), were infrequent and managed conservatively. Overall, RIA-MIND demonstrates a favorable safety profile comparable to, or better than, conventional OLND.

### 5.3 Oncological adequacy and nodal yield

Oncological efficacy was assessed using LN yield as a surrogate marker. The median ipsilateral (25.00) and contralateral yield (17.00) are consistent with accepted thresholds for adequate oncologic staging and treatment. Previous studies in elective neck dissection have shown that retrieval of approximately 18 LNs is sufficient for accurate pathological staging, with minimal incremental survival benefit beyond this number (18), while robotic and endoscopic series have reported comparable nodal yields with limited nodal positivity (19). In accordance with UICC and AJCC recommendations, a minimum of 6 LNs is required for adequate pathological evaluation (20, 21). In the present series, all but 1 ipsilateral dissection yielded ≥12 LNs, and all but 1 contralateral dissection yielded ≥10 LNs, thereby meeting accepted criteria for oncologic adequacy. These findings indicate that RIA-MIND consistently achieves nodal yields aligned with international oncologic standards.

### 5.4 Learning curve analysis using CUSUM

#### 5.4.1 Operative efficiency and learning phases

Mean-referenced CUSUM analysis of operative (console) time demonstrated a clear learning trajectory during sequential RIA-MIND procedures. The CUSUM curve showed an initial upward trend during the early cases, indicating console times above the cohort mean during the learning phase. This was followed by a downward trajectory during the transition phase and a sustained decline in later cases, reflecting progressive improvement and stabilization of console efficiency with increasing experience. Segmented regression identified two inflection points at cases 13 and 27, defining distinct learning (cases 1–13), transition (cases 14–27), and proficiency (cases ≥28) phases.

Although formal CUSUM-based learning curve analyses are limited in the head and neck robotic literature, the learning pattern observed in the present study is consistent with trends reported for other robotic head and neck procedures. A retroauricular robot/endoscope-assisted neck dissection study demonstrated a progressive reduction in operative time with increasing experience in robot-assisted neck dissection, attributed to improved familiarity with robotic logistics and operating room coordination (19). Similarly, a 3-year prospective case study reported a significant and gradual decrease in operative time across experience-based subgroups in both modified radical and selective robotic neck dissections, without adverse effects on other perioperative parameters (22). In a 4-year prospective analysis, the authors described a steep early learning curve followed by a plateau phase, highlighting a reduction in operative time as a key marker of procedural maturation (23). The close alignment between these studies and the present CUSUM-based analysis supports the validity of the learning phases identified for RIA-MIND.

RIA-MIND represents an innovative robotic approach that utilizes a novel infraclavicular working corridor; therefore, its learning curve reflects early institutional experience rather than that of a mature, widely standardized technique. The structured learning pattern observed suggests that the approach is reproducible; however, as emphasized by a 4-year prospective analysis, learning trajectories are inherently influenced by surgeon experience, team familiarity, case volume, and institutional workflow (23). Consequently, although the absolute case thresholds may not be directly generalizable, a similar trajectory of progressive efficiency with experience may be anticipated as the technique is adopted more broadly.

#### 5.4.2 Oncological consistency and nodal yield

Mean-referenced CUSUM analysis of ipsilateral nodal yield demonstrated a clear learning trajectory, with early negative deviation from the mean followed by a sustained upward trend in later cases. Segmented regression identified distinct learning, transition, and proficiency phases, with nodal retrieval consistently exceeding the cohort mean in the proficiency phase, indicating progressive stabilization of oncological adequacy with increasing experience. Similar trends have been reported in robotic and endoscopic neck dissection literature, where refinement of robotic technique has been closely linked to oncologic metrics such as nodal yield, while stable oncologic outcomes are maintained across learning phases despite gains in operative efficiency (19, 22).

#### 5.4.3 Perioperative complications

Binary (Bernoulli) CUSUM analysis demonstrated early fluctuations in perioperative complications followed by stabilization without a sustained upward trend, indicating that perioperative safety was maintained throughout the learning process. Although a progressive reduction in complication rates after attainment of proficiency was not observed in the present study, the stable CUSUM profile suggests that increasing surgical experience was not associated with deterioration in patient safety. Similar learning patterns have been reported in previous robotic neck surgery series, where perioperative safety was preserved while operative efficiency improved with experience (23, 24).

#### 5.4.4 Methodological strength and benchmarking

A key strength of this study is the concurrent application of CUSUM methodology to console time, nodal yield, and complications. CUSUM offers an objective framework for defining learning curves in robotic surgery, with learning thresholds typically ranging from 15 to 40 cases and remaining surgeon-specific (25, 26). By integrating learning curves across multiple performance domains, this study provides a multidimensional assessment of the RIA-MIND learning process. It establishes a structured benchmark for future evaluation of this novel robotic technique.

### 5.5 Limitations

This study has several limitations. Its retrospective design and single-center setting may limit the generalizability of the findings. Patient selection for the RIA-MIND approach was based on clinical suitability and patient preference, reflecting routine clinical practice and introducing the potential for selection bias. Although the sample size was sufficient to demonstrate the feasibility and safety of the technique, it remains relatively modest. The follow-up duration was insufficient to assess long-term oncologic outcomes, functional recovery, cosmetic outcomes, and quality of life. Moreover, the present study was designed primarily to evaluate peri-operative feasibility, safety, and early postoperative outcomes rather than long-term oncologic survival; therefore, formal survival analysis was beyond the intended scope of the study.

Objective assessment of shoulder function, validated patient-reported outcome measures, and standardized cosmetic evaluation were not routinely collected and therefore could not be analyzed. All procedures were performed by a single surgeon with prior experience in robotic surgery, including surface-insufflation procedures in other surgical specialties. This prior robotic experience may have influenced the observed learning curve and proficiency thresholds, which should therefore be interpreted as surgeon- and setting-specific rather than universally generalizable. The absence of a matched open lateral neck dissection (OLND) or alternative robotic control group further precludes definitive conclusions regarding comparative effectiveness, superiority, or non-inferiority. Future prospective, multicenter comparative studies involving surgeons with varying levels of robotic experience and incorporating long-term oncologic, functional, and cosmetic outcomes are warranted to further validate the RIA-MIND approach.

## 6 Conclusion

RIA-MIND is a feasible and safe remote-access alternative to conventional OLND in selected head and neck cancers, enabling comprehensive cervical nodal clearance through cosmetically favorable infraclavicular incisions. The technique provides satisfactory nodal yield, facilitates preservation of key neurovascular structures, and is associated with low perioperative morbidity. CUSUM analysis demonstrated a structured learning curve, with progressive improvement in console efficiency, stabilization of nodal yield, and maintenance of perioperative safety over sequential cases. Collectively, these findings support RIA-MIND as a promising minimally invasive approach to neck dissection and provide a foundation for further validation in prospective multicenter studies with long-term oncologic, functional, and cosmetic outcomes.

## Data Availability

The raw data supporting the conclusions of this article will be made available by the authors, without undue reservation.
